# POSITIVE AFFECT IN DEMENTIA CARE DYADS

**DOI:** 10.1093/geroni/igac059.1573

**Published:** 2022-12-20

**Authors:** Joan Monin, Claudia Haase

## Abstract

Positive affect is important for physical health, well-being, and relationships. Yet, most studies of dementia care dyads have focused on negative outcomes, such as depression and caregiver burden. We present findings from studies that address this important research gap. The first two speakers present findings from actor partner interdependence models using secondary data from a randomized controlled trial of persons with early-stage dementia (PWD) and their spouses. Specifically, Dr. Monin will show that actor quality of life is significantly related to positive affect cross-sectionally, controlling for functional status of each partner and PWD behavioral symptoms. Ms. Piechota will present longitudinal findings demonstrating that when spouses were high in difficulty regulating emotions, the PWD’s positive affect decreased over three months, controlling for intervention arm and covariates. The second two speakers will present findings from two different studies of persons living with behavioral-variant frontotemporal dementia (bvFTD), Alzheimer’s disease (AD), and controls that engaged in laboratory conflict discussions. Dr. Brown will show that caregivers of individuals with bvFTD reported greater decreases in positive emotion across the discussion relative to caregivers of individuals with AD and language variants. She will also discuss how caregivers who reported greater decreases in positive emotion had higher levels of depressive symptoms, controlling for their partner’s diagnosis and level of cognitive impairment. Finally, Dr. Chen will provide evidence that bvFTD caregivers had lower emotional well-being according to the SF-36 than AD caregivers and controls, and this effect was fully mediated by bvFTD caregivers' lower positive emotional connections.

